# Development and content validity of an application to assess 24-hour movement behaviors in 0–4-year-old children involving end-users and key stakeholders: the My Little Moves app

**DOI:** 10.1186/s12966-023-01552-9

**Published:** 2024-01-02

**Authors:** Jelle Arts, Mai J. M. Chinapaw, Jessica S. Gubbels, Arnoud P. Verhoeff, Annette Brons, Sanne Veldman, Annelinde Lettink, Teatske M. Altenburg

**Affiliations:** 1 Public and Occupational Health, Amsterdam UMC location Vrije Universiteit Amsterdam, De Boelelaan 1117, Amsterdam, The Netherlands; 2 Health Behaviors and Chronic Diseases, Amsterdam Public Health, Amsterdam, The Netherlands; 3 Methodology, Amsterdam Public Health, Amsterdam, The Netherlands; 4 Department of Health Promotion, NUTRIM School of Nutrition and Translational Research in Metabolism, Maastricht University, PO Box 616, Maastricht, 6200 MD The Netherlands; 5 Public Health Service Amsterdam, Sarphati Amsterdam, 1018 WT Amsterdam, The Netherlands; 6 Department of Sociology, University of Amsterdam, 1018 WV Amsterdam, The Netherlands; 7 Digital Life Center, Amsterdam University of Applied Sciences, Wibautstraat 3B, Amsterdam, 1091 GM The Netherlands

**Keywords:** Physical activity, Sedentary behavior, Sleep, Early childhood, Parent-report, Mobile app, Content validity

## Abstract

**Background:**

Recently, research focus has shifted to the combination of all 24-h movement behaviors (physical activity, sedentary behavior and sleep) instead of each behavior separately. Yet, no reliable and valid proxy-report tools exist to assess all these behaviors in 0–4-year-old children. By involving end-users (parents) and key stakeholders (researchers, professionals working with young children), this mixed-methods study aimed to 1) develop a mobile application (app)-based proxy-report tool to assess 24-h movement behaviors in 0–4-year-olds, and 2) examine its content validity.

**Methods:**

First, we used concept mapping to identify activities 0–4-year-olds engage in. Parents (*n* = 58) and professionals working with young children (*n* = 21) generated a list of activities, sorted related activities, and rated the frequency children perform these activities. Second, using multidimensional scaling and cluster analysis, we created activity categories based on the sorted activities of the participants. Third, we developed the My Little Moves app in collaboration with a software developer. Finally, we examined the content validity of the app with parents (*n* = 14) and researchers (*n* = 6) using focus groups and individual interviews.

**Results:**

The app has a time-use format in which parents proxy-report the activities of their child, using eight activity categories: personal care, eating/drinking, active transport, passive transport, playing, screen use, sitting/lying calmly, and sleeping. Categories are clarified by providing examples of children’s activities. Additionally, 1–4 follow-up questions collect information on intensity (e.g., active or calm), posture, and/or context (e.g., location) of the activity. Parents and researchers considered filling in the app as feasible, taking 10–30 min per day. The activity categories were considered comprehensive, but alternative examples for several activity categories were suggested to increase the comprehensibility and relevance. Some follow-up questions were considered less relevant. These suggestions were adopted in the second version of the My Little Moves app.

**Conclusions:**

Involving end-users and key stakeholders in the development of the My Little Moves app resulted in a tailored tool to assess 24-h movement behaviors in 0–4-year-olds with adequate content validity. Future studies are needed to evaluate other measurement properties of the app.

**Supplementary Information:**

The online version contains supplementary material available at 10.1186/s12966-023-01552-9.

## Background

A healthy combination of all 24-h movement behaviors—encompassing physical activity, sedentary behavior, and sleep – supports the growth and development of young children [1–4]. Therefore, the World Health Organization and several countries developed 24-h movement guidelines for infants (0–1 year), toddlers (1–3 years) and preschoolers (3–5 years) [5–8]. Unfortunately, the quality of evidence supporting these guidelines is considered low [5, 9]. A significant factor contributing to the lack of high-quality research on young children’s 24-h movement behaviors is the difficulty of accurately measuring these behaviors among this age group [9].

Adequate assessment of 24-h movement behaviors in young children requires affordable, feasible, valid and reliable measurement instruments, adapted to the child’s developmental stage. Accelerometers are widely considered as a promising method for assessing 24-h movement behaviors, as they can capture data on body movement continuously over extended periods of time. Although accelerometers are considered valid and reliable for measuring 24-h movement behaviors in children from preschool age [10–13], its validity for infants and toddlers is yet to be established [13, 14]. Additionally, there is currently no consensus about the optimal measurement protocol (e.g., wearing location) and accelerometer data processing decisions (e.g., definition of non-wear time, choice of cut-points or algorithms to classify physical activity, sedentary behavior or sleep) for the use of accelerometers in young children [13]. Moreover, current data processing procedures do not take into account that accelerometer output in very young children may reflect movements of others, e.g., parents carrying their child [15]. Beside accelerometers, direct observation is considered an accurate measure of movement behaviors in children [16, 17]. However, observation is labor intensive and intrusive, and thereby not feasible to use on a large scale and/or for a longer period.

Alternatively, proxy-report tools such as parent-reported questionnaires or diaries can be used to assess young children’s 24-h movement behaviors. These tools can be used in large samples, in a relatively convenient and affordable way, with the additional advantage of obtaining information about the type (e.g., screen time) and context (e.g., location) of the behavior [18]. A number of proxy-report tools have been developed to assess physical activity, sedentary behavior and/or sleep in early childhood, though currently no reliable and valid tools exist to assess all 24-h movement behaviors in 0–4-year-old children [19, 20].

The lack of valid and reliable proxy-report tools can be explained by limitations of questionnaires and diaries in general, such as social desirability and recall bias [18]. In addition, young children’s sporadic and intermittent behaviors may be particularly difficult to summarize in a proxy-report. Another possible explanation may be the lack of involvement of end-users in the development of proxy-report tools [19]. Consequently, it remains unclear whether end-users (e.g., parents of young children) consider the content of such proxy-report tools as relevant, comprehensive and comprehensible, and whether it is feasible for them to complete the tool. Evaluating content validity is an important first step in ensuring that proxy-report tools measure what they intend to measure [21]. Given that lacking content validity can affect all other measurement properties [21, 22], it is essential to engage end-users in the development of questionnaires or diaries.

Furthermore, existing proxy-report tools for assessing young children’s 24-h movement behaviors are often not tailored to a specific age group (e.g., infants, toddlers or preschoolers) or developmental stage [19]. However, during this period of rapid (motor) development, movement behaviors are very different between different ages, e.g., daytime naps and tummy time in infants versus running and cycling in preschool aged children [5]. For this reason, tailored proxy-report tools are needed that assess developmentally appropriate activities.

Online assessment tools, such as web-based questionnaires or diaries, may offer advantages over paper-based tools in assessing movement behaviors. For example, online tools might be easier to administer, and the online interface can be used to tailor the questions or format to the developmental stage of each child (e.g., hide irrelevant activities or questions) [23]. Compared to web-based questionnaires or diaries, mobile applications (apps) may be even more beneficial for parents as they often carry a mobile device, which enables reporting movement behaviors in real-time [24, 25].

By involving end-users (parents) and key stakeholders (researchers, professionals working with young children), this mixed-methods study aimed to 1) develop a mobile app-based proxy-report tool to assess 24-h movement behaviors in 0–4-year-old children, and 2) examine its content validity.

## Methods

### General procedures

We developed a mobile app called ‘My Little Moves’ following the COnsensus-based Standards for the selection of health Measurement INstruments (COSMIN) methodology for content validity [21]. In this process, five steps were completed (Table 1). In step 1, we used concept mapping to explore the activities that 0- to 4-year-old children engage in. In step 2, we designed and developed the My Little Moves app in collaboration with software developer Eaglescience. In step 3, the content validity of the My Little Moves app was evaluated among parents and researchers. Here, online focus group- and individual interviews were held to explore the comprehensiveness, comprehensibility, relevance, user friendliness and feasibility of the app [21]. In step 4, based on the results of step 3, the My Little Moves app was adapted. In step 5, the adapted version of the My Little Moves app was evaluated among parents using online individual interviews.

**Table 1:**
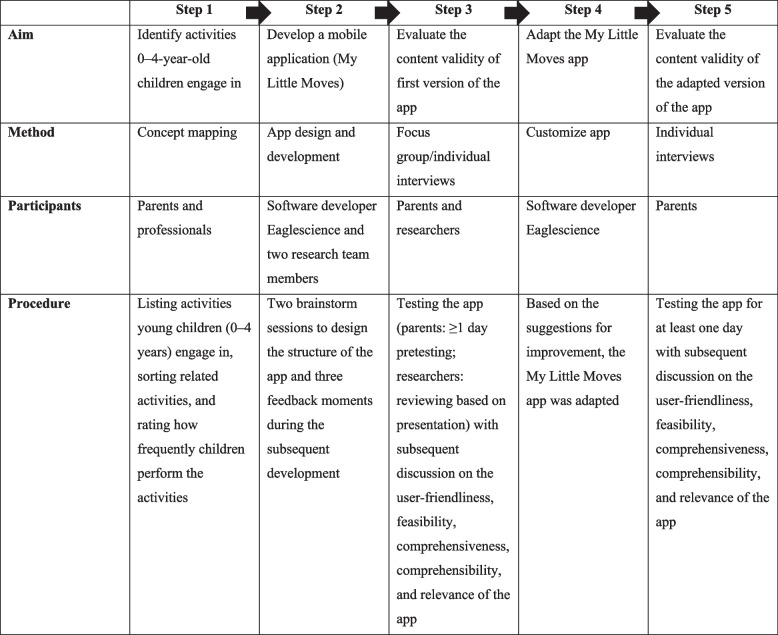
Overview of the steps undertaken to create the My Little Moves app to assess 24-h movement behaviors in 0–4-year-old children

### Participants and recruitment

For steps 1, 3 and 5, parents of apparently healthy children aged 0–4 years old were recruited. Apparently healthy children were defined as: typically developing children born term (> 37 weeks) and without developmental disorders or any medical diagnoses. For step 1, professionals working with young children were also included, e.g. early childhood education and care (ECEC) professionals, youth health care professionals (e.g., youth health care physicians), and pediatric physiotherapists. For step 3, researchers with expertise in measurement instrument development and evaluation and/or physical activity, sedentary behavior and sleep in young children were included in addition to parents. In step 1 and step 3, most participating parents had a high educational degree (see “Results” section). Therefore, in step 5, to ensure the user-friendliness of the app among parents with lower educational degrees, only parents with a maximum educational level of general secondary education or vocational education were included, excluding parents with a bachelor’s or master’s degree. As the app is in Dutch, all participants had to be able to read the Dutch language.

ECEC centers and child health services in the Netherlands were approached by email for study recruitment, and in case of agreement asked to send an information letter to parents and professionals. In addition, parents, professionals, and researchers were recruited through the personal network of the research team and the network of the project consortium. Social media was also used to recruit parents. Informed consent was obtained from all participants before study participation. The Amsterdam UMC Medical Ethical Committee approved the study protocols (nrs. 2020.334, 2021.0758).

### Step 1: Concept mapping

#### Design

Concept mapping is a mixed method for identifying and visualizing group ideas on a particular topic, combining qualitative data collection and subsequent qualitative and quantitative data analysis [26, 27]. Separate concept mapping sessions were organized to identify activities for the following age categories: children aged up to 6 months, 6 to 12 months, 12 to 24 months, 24 to 36 months, and 36 to 48 months. These categories were chosen after consulting professionals working with children aged 0–4 years old (i.e., youth health care physicians and pediatric physiotherapists, *n* = 5) in the network of our research project consortium by phone and email. These professionals emphasized that, due to differences in motor development, the activities these children engage in should be discussed in separate sessions.

We aimed to recruit at least 10 parents and professionals for each session targeting the above-mentioned age categories. Groups of at least 10 participants promote a sufficient variety of ideas [27]. Participants could participate in concept mapping sessions for more than one age group (i.e., when parents had multiple children aged below 4 years).

#### Procedures

Five concept mapping phases were conducted from December 2020 to June 2021: 1) individual idea generation towards a focus statement; 2) combining ideas and adding new ideas in a group session; 3) individual sorting and rating of the generated ideas; 4) statistical analysis; and 5) interpretation of the concept maps. Concept mapping sessions were performed online due to COVID-19-related restrictions of face-to-face contact at the time in the Netherlands. While phase 1 and 2 are usually combined during face-to-face concept mapping sessions, we separated these phases due to the online format of our concept mapping sessions.

In the first phase, participants were invited to participate in a survey using Survalyzer. This survey started with collecting information on participants’ characteristics, including gender, age, and the country of birth of the participants and their parents. For parents, also their highest educational level and age of their children was collected, and for professionals their profession and number of years of experience. Next, to get familiar with the method, participants were asked to think about activities of children in the specific age group, by answering the following warming up question: ‘What activities do children aged … months/years enjoy doing?’. Subsequently, each participant was asked to generate as many activities as possible based on the main focus statements:

‘The activities that children aged … months engage in during a day (24 hours) are:…’

‘What are the activities that children aged … months engage in during a day (24 hours)?’

In the second phase, the research team (JA, JG, SV and TA) combined all ideas, deleted duplicate ideas and subsequently made a list of all unique activities. Hereafter, participants were invited to join an online group brainstorm. In this online meeting, two researchers (JA and SV) presented the combined list of activities, and participants checked the clarity of the activities on the list. Next, the participants could add additional activities when they were inspired by other mentioned activities. Participants who were not able to attend an online group brainstorm received the combined list of activities by email, and were also asked to check all listed activities on clarity and were given the opportunity to add new activities. Based on the comments and additions of participants, a final list of unique activities was generated for each age group. The second phase was completed when no new activities were mentioned by the participants.

In the third phase, participants received a link to a self-developed web application [28], in which the list of unique activities generated in phase 1 was presented. Using this application, the participants sorted the activities into groups of related activities, and subsequently named the groups. Next, the participants rated the frequency of all individual activities among children of each specific age group using a five-point Likert-scale, ranging from never (1) to very often (5).

During the fourth phase, R-CMap was used as a statistical tool to create the concept maps from the sorted activities [29]. R-CMap is open-source software for concept mapping, available in R, of which version 3.6.3 was used. Five concept maps (one for each age group) were created using a multidimensional scaling algorithm and hierarchical cluster analysis. In these concept maps, activities were shown as a point on a figure, with activities sorted more often together appearing closer to each other and activities never/rarely sorted together widely separated, thereby forming clusters of activities. Each cluster represents a group of activities of a similar concept based on the participants’ sorting.

In the last phase, researchers with multiple years of experience in young children’s movement behaviors (JA, TA, JG and SV) discussed the meaning of the different concept maps, and analyzed the optimal number of clusters to represent the participant’s ideas (i.e., activities). After defining a final number of clusters, the researchers named the clusters based on the names given by the participants in the third phase, with each cluster representing a specific activity category. Some of the activities were moved between clusters if, based upon the researchers’ perspective, they fitted better within another activity category. Based on the frequency rating, average ratings for each activity and each cluster were calculated.

### Step 2: Design of the My Little Moves app

From August 2021 to January 2022, software developer Eaglescience designed and developed the My Little Moves app. The main activity categories (i.e., clusters of activities) identified in Step 1 (concept mapping study) were used as the basis for the design. This procedure was inspired by the development of MyDailyMoves: an online tool to assess the 24-h movement behaviors of 9–12-year-old children [30].

The software development team (including three developers and one design specialist) and two researchers (JA and TA) brainstormed in two sessions on the design of the app. The first brainstorm, focused on ideas for the design of the app and requests of the research team, including ‘must have’ and ‘nice to have’ features. Based on the input of this session, the design specialist created wireframes for the structure and lay-out of the app. These wireframes were discussed in the second brainstorm session. After the brainstorm phase, the research team (JA, MC, JG, AV, SV, and TA) decided on the final design of the My Little Moves app. Subsequently, the app was developed in three phases of approximately four weeks, with two researchers (JA and TA) giving feedback after each phase.

### Step 3: Evaluating the content validity of first version of My Little Moves

From February to March 2022, the My Little Moves app was pilot-tested among parents and researchers. In line with the COSMIN criteria, we aimed to include at least 15 parents and 5 researchers [21]. After giving informed consent, we collected information on gender and country of birth from all participants. From parents, we collected additional information about their age, highest educational level and the age group of their child(ren), and from researchers information about their expertise, profession and number of years of experience. Subsequently, each participant received a unique login code by email to download and use the app. Parents were asked to complete the app for at least one day, to obtain their first experiences with using the app. Researchers were asked to go through and reflect on the app. After testing the My Little Moves app, participants were invited to join an online focus group to evaluate the app. Focus groups were scheduled based on the availability of participants. When it was not possible for participants to join a focus group an individual online interview was scheduled.

Two trained facilitators (JA and either SV or AL) discussed the app together with the participants using a structured interview guide. After discussing the first impression of the app, each item was discussed in-depth regarding user-friendliness, feasibility, relevance, comprehensiveness and comprehensibility. All sessions were recorded using a voice recorder. Recordings of all sessions were transcribed verbatim, for analyses. Finally, the limitations and suggestions for improvement were extracted by one researcher (JA).

### Step 4: Adaptation of My Little Moves

Five researchers (JA, MC, JG, SV, and TA) discussed the identified limitations and suggestions for improvement of the My Little Moves app, and decided on the final adaptations taking into account the available budget for the development of the app. The My Little Moves app was adapted accordingly in April 2022.

### Step 5: Evaluating the content validity of adapted version of My Little Moves

From May to June 2022, the adapted version of the My Little Moves app was pilot-tested and discussed during online individual interviews with parents. We aimed to include at least 10 parents with maximally secondary education or vocational education. A facilitator (student medical informatics) trained by a researcher (JA) discussed the user-friendliness, feasibility, relevance, comprehensiveness and comprehensibility of the adapted app together with the participants using a structured interview guide. All interviews were recorded using a voice recorder, and the recordings were transcribed verbatim. Finally, one researcher (JA) and the facilitator independently extracted limitations and suggestions for improvement.

## Results

### Step 1: Concept mapping

#### Participants

In total, 95 parents and 26 professionals signed informed consent to participate in the concept mapping study. In the first phase, 61 parents (64.2%) and 21 professionals (80.8%) participated. Table 2 shows the socio-demographic characteristics of the participants. The majority of parents were highly educated, with 51 parents having a master’s degree. Of the professionals that participated in the first concept mapping session, 16 were pediatric physiotherapists, two were ECEC professionals, two were youth health care physicians, and one was a pedagogical policy officer. In the second phase, 22 parents and 14 professionals participated, and in the third phase 31 parents and 15 professionals participated. Characteristics of participants were similar across the concept mapping phases.

**Table 2:** Socio-demographic characteristics of participants in the first concept mapping session

	**Parents**					**Professionals**				
**Age group**	0–6	6–12	12–24	24–36	36–48	0–6	6–12	12–24	24–36	36–48
**Total number**	6	8	16	13	18	6	5	3	5	2
**Mean age ± SD (years)**	33.2 ± 2.0	32.6 ± 1.4	33.5 ± 3.1	33.5 ± 3.8	34.5 ± 3.2^*^	43.2 ± 13.4^*^	46.4 ± 13.4	35.7 ± 8.3	45.4 ± 9.8	35.5 ± 3.5
**Gender**										
Female / Male	5 / 1	8 / 0	15 / 1	13 / 0	17 / 0^*^	4 / 1^*^	5 / 0	3 / 0	5 / 0	2 / 0
**Highest educational level**										
SED or VOC ED	0	0	4	1	3	0	1	0	1	0
Bachelor’s degree	0	0	0	0	3	1	1	1	2	0
Master’s degree	6	8	12	12	12	5	3	2	2	2
**Country of birth participant**										
Netherlands	6	8	15	12	17^*^	6	5	3	5	2
Other	0	0	1	1	0	0	0	0	0	0
**Country of birth father**^**a**^										
Netherlands	3	6	15	11	15	5	5	3	4	2
Other	3	2	1	2	2	1	0	0	1	0
**Country of birth mother**^**a**^										
Netherlands	5	6	15	12	16	6	5	2	5	2
Other	1	2	1	1	1	0	0	1	0	0
**Profession**										
Pediatric physiotherapist	NA	NA	NA	NA	NA	4	3	3	4	2
Pediatric policy officer						1	0	0	0	0
Youth health care physician						1	1	0	0	0
ECEC professional						0	1	0	1	0

*Abbreviations: ECEC* Early childhood education and care, *SED* Secondary education, *NA* not applicable, *VOC ED* Vocational education

^*^Not reported by one participant

^a^Country of birth of the father and mother of the participant, i.e., grandfather and grandmother of their child

#### Concept maps

The participants collectively listed between 110 and 136 unique activities per age group (i.e., 0–6, 6–12, 12–24, 24–36, and 36–48 months). From these activities, five final concept maps were created (one for each age group) ranging from seven to nine clusters, reflecting different activity categories. Table 3 shows the activity categories obtained from the concept maps, and the average frequency ratings for each activity category. For the age group 0–6 months, we did not find clearly matching clusters based on the hierarchical cluster analysis. Therefore, for this age group researchers (JA, TA, JG and SV) created activity categories based on the activity categories for the older age groups and the activities listed by parents and professionals. For the other age groups, the research team added a few activity categories to better represent the listed activities (underlined in Table 3). Average frequency ratings of the activity categories ranged from 2.1 for the category screen use (age group 6–12 months) to 4.7 for the category sleeping (age group 12–24 months). Additional file 1 presents activity categories for each age group, as well as the average frequency ratings of the individual activities. Based on the activity categories for all age groups, the following activity categories were included in the My Little Moves app: personal care, sitting/lying calmly, active transport, passive transport, playing, screen use, and sleeping.

**Table 3:** Activity categories of children in the age groups 0–6 months, 6–12 months, 12–24 months, 24–36 months and 36–48 months, sorted by frequency^a^

Age group	Activity category (average frequency rating ± mean SD)	Examples of activities
**0–6 months**	Play with objects or own hands (3.85 ± 0.74)	Manipulating toys, playing with toy, playing with hands, hitting with toys
	Calm activities (3.63 ± 0.77)	Listening to music, watching on screens (television, mobile phone)
	Play in prone position (3.61 ± 0.89)	Lying on the tummy, reaching for objects in the prone position, raising the head in the prone position, playing on the tummy with toy
	Sleeping (3.56 ± 0.64)	Sleeping, sleeping in the baby carrier
	Care/activities of daily living (3.57 ± 0.75)	Being cared for (applying cream, brushing teeth), eating, crying, peeing/pooing, being breastfeed, drinking bottle, dressing/undressing, taking a bath
	Play in supine position (3.48 ± 0.76)	Playing with toy on chest in supine position, hitting toys in baby gym, putting feet up/sideways on back, playing with the feet (grasping feet in the supine position, feet-hands game)
	Restrained sitting/lying (3.47 ± 0.81)	Sitting in a chair, lying in the pram, sitting in a bouncer, being carried upright, sitting in car seat, lying in the baby carrier
	Interactive play (3.34 ± 0.89)	Singing (together), being tickled, playing on lap, playing ‘airplane’, having a book read aloud, playing peek-a-boo, imitating/mirroring
	Maintaining posture/postural transitions (3.27 ± 0.84)	Crawling, pulling up, rolling over to side position, kick feet, swinging arms, reaching, grab, rocking, controlled movement of head/neck
**6–12 months**	Sleeping (4.37 ± 0.70)	Sleeping, taking morning/afternoon nap, falling asleep/lying quietly in bed
	Care/activities of daily living (3.60 ± 0.75)	Changing diaper, taking a bath, drinking a bottle with help, going outside, eating without help, peeing/pooing, being breastfed
	Calm activities (3.35 ± 0.83)	Crowing/babbling, drooling, sitting in bouncer, lying on parent’s stomach, laughing
	Play with objects or own hands (3.29 ± 0.91)	Manipulating objects in the hands, putting materials in the mouth, drawing/coloring, pulling string, touching parent’s face, clapping hands, feeling different fabrics
	Passive transport (3.28 ± 0.93)	Sitting in car, sitting on the bicycle/in a bicycle trailer, being carried in a hiking backpack, being carried
	Maintaining posture/postural transitions (3.24 ± 1.02)	Pushing up to sit, standing with support, rolling from tummy to back, lying on the side, rolling from back to tummy
	Active transport (2.76 ± 1.13)	Climbing/clambering, shuffling buttocks, walking with support, crawling, grabbing feet, walking on the balance bike
	Play (2.72 ± 0.94)	Rolling a ball, drawing/coloring, playing peek-a-boo, rocking in a baby swing, reading a book
	Screen use (2.11 ± 0.95)	Watching on the tablet, watching TV, looking at the phone
**12–24 months**	Sleeping (4.67 ± 0.49)	Sleeping, taking morning nap/afternoon nap
	Care/activities of daily living (4.09 ± 0.86)	Brushing teeth, going to the toilet/potty, taking a shower, changing a diaper, eating, dressing/undressing, drinking
	Calm play (3.74 ± 0.77)	Reading a book, listening to music, playing quietly with toys (shape sorter, Duplo, toys with sounds)
	Interactive play (3.69 ± 0.94)	Playing catch/tag, playing hide and seek, playing with friends/sister/brother, playing with animals
	Calm creative play (3.51 ± 0.93)	Coloring/scratching with pencil/drawing/stamping, building with blocks/building tower, making music, doing puzzles
	Active play (3.45 ± 0.89)	Rolling a ball, playing in the playground, playing in the sandpit, swinging, playing football, jumping on a trampoline
	Active transport (3.33 ± 1.01)	Crawling, bicycling, walking backwards, climbing/clambering, crouching, walking behind a cart, walking, running
	Passive transport/sedentary activities (3.21 ± 1.01)	Sitting (on sofa, stairs, chair), sitting in a car, sitting in a stroller, sitting in a bicycle seat
	Screen use (2.70 ± 1.21)	Watching TV, watching on a tablet/playing games/scrolling, looking at a phone/playing games/scrolling
**24–36 months**	Sleeping (4.11 ± 0.93)	Sleeping, taking a short sleep/afternoon nap
	Interactive play (3.73 ± 0.71)	Hugging/petting animals, being tickled, playing together, imitating others, playing peek-a-boo, helping with household chores
	Care/activities of daily living (3.68 ± 0.95)	Showering, taking a bath, using the potty/going to the toilet, brushing teeth, changing diaper, drinking
	Calm creative play (3.47 ± 0.74)	Building with Lego/Duplo/blocks, reading book, doing puzzles, tinkering, drawing/coloring
	Screen use (3.39 ± 0.83)	Playing games on iPad/tablet, watching TV/Netflix/tablet movies
	Active play (3.30 ± 0.81)	Playing in the playground, flying a kite, swinging, jumping on the trampoline, doing hide and seek, playing with a ball
	Passive transport (2.96 ± 0.90)	Sitting on a bicycle seat, sitting in the car, sitting in the buggy
**36–48 months**	Care/activities of daily living (3.85 ± 0.72)	Washing hands, showering, going to the toilet, dressing/undressing, brushing your teeth, eating, drinking
	Interactive play (3.53 ± 0.91)	Playing with pets, playing interactive games, helping in the garden, playing with other children, imitating movements
	Outdoor play (3.48 ± 0.70)	Playing with water, playing in the woods, visiting amusement parks/zoos/petting zoo/beach, playing in a playground, playing in the sandbox
	Sleeping (3.45 ± 1.02)	Sleeping, taking an afternoon nap
	Calm play (3.18 ± 0.83)	Stringing beads, making music, playing with Playmobil, coloring/drawing/painting, tinkering, building a marble run, doing puzzles
	Active play (3.05 ± 0.93)	Playing with sports equipment, sliding, dancing, playing with a ball, throwing objects, running, jumping
	Passive transport (2.71 ± 0.91)	Sitting in the buggy, sitting on a bicycle seat, using public transport, sitting in a car seat
	Screen use (2.33 ± 0.83)	Playing games on tablet/iPad/phone, watching TV/Netflix/Disney/DVD, playing games on a game console

Underlined activity categories were added by the research team to better represent the listed activities

^a^Rated by parents and professionals on a 5-point Likert-scale ranging from never (1) to very often (5)

### Step 2: Design of the My Little Moves app

#### Format

**Fig. 1:**
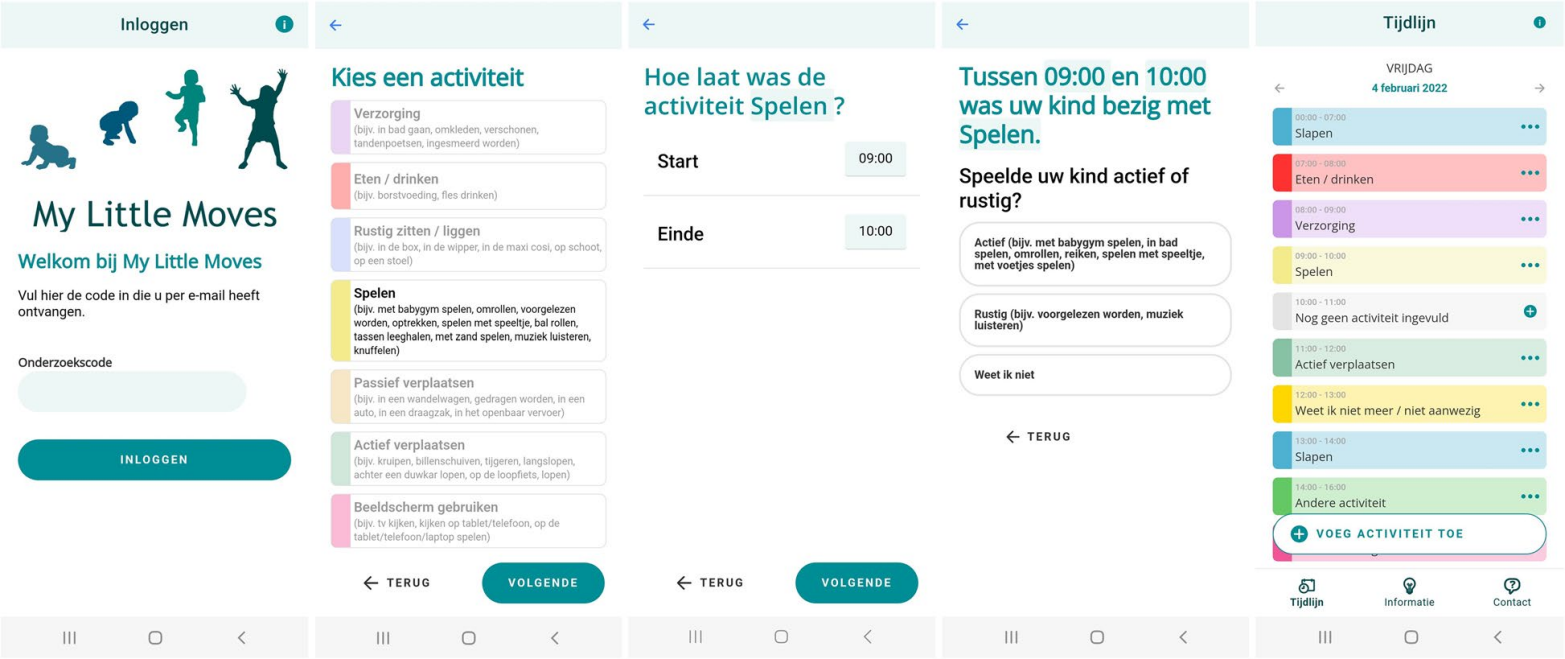
Screenshots of the first version of the My Little Moves app. From left to right: login screen; choosing an activity category, with age-appropriate activity examples; selecting the time of the activity; follow-up question of the activity category ‘playing’: ‘*Did your child play actively or calmly’*; timeline, entered activities in the daily time-use overview

My Little Moves is an app in Dutch that can be used on smartphones and tablets, and can be downloaded for free in Google Play (Android version 5.1 or later) and the App Store (iOS version 13.0 or later). After installation of the app, parents can create an account with a personalized code. To increase data security, parents must create a password. Parents remain logged in for 7 days, except for when they actively log out via a button in the app. After 7 days, parents are automatically logged out. The format consists of a time-use diary. We chose this format as studies indicate that a time-use diary provides more accurate estimates of young children’s behaviors than a recall questionnaire [19, 31]. In the My Little Moves app, activities are reported per day, from 00:00 to 23:55 (not 24:00 for technical reasons), for 7 consecutive days. The activities are reported sequentially, with the end time of the previous activity automatically indicating the start time of the next activity. Figure 1 presents screenshots of the first version of the My Little moves app.

**Fig. 2:**
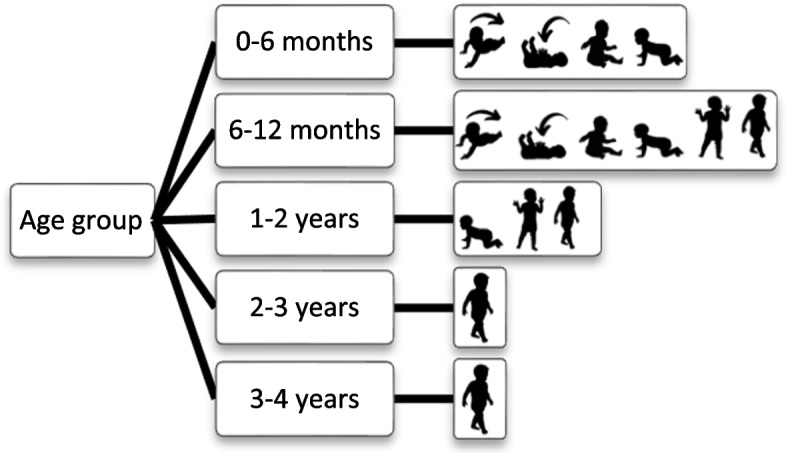
Motor development milestones assessed in the My Little Moves app per age group: if child is able to 1) roll over from the belly to the back and 2) from the back to the belly, 3) sit for at least five seconds without support, 4) crawl for at least 1.5 m, 5) stand for at least five seconds without support, and 6) walk three steps without support

The app is tailored to the age and motor development of the child. When parents use the app for the first time, the following questions are asked to assess the child’s developmental stage: age group of the child (0–6 months, 6–12 months; 1–2 years, 2–3 years or 3–4 years) and 1–6 questions on achievement of motor milestones relevant for the selected age group (if motor milestone was achieved, and if yes, at which age). Included motor milestones were 1) roll over from back to belly, 2) roll over from belly to back, 3) sit without support, 4) crawl, 5) stand without support, and/or 6) walk without support. Figure 2 shows the milestones assessed per age group [32].

#### Content

The first version of the My Little Moves app included the following activity categories: 1) personal care, 2) eating/drinking, 3) sitting/lying calmly, 4) active transport 5) passive transport, 6) playing, 7) screen use, 8) sleeping, 9) other activity, 10) I don’t remember/not present. The category ‘eating/drinking’ did not result from the concept maps, but was added by the research team as we considered it useful to assess these frequently occurring activities (frequency ratings ranged from 4.0 to 4.8) separately. For each of the activity categories, age-appropriate activity examples from the concept maps were included. We included the category ‘I don’t remember/not present’ as parents are not always present to report the activities of their child, or because they might forget what activity their child did. In addition, we included the category ‘other activity’ to allow parents to report activities that – according to them—do not belong to any of the other categories.

To add an activity to the timeline, parents first choose an activity category and thereafter select the start and end time of the activity with 5 min-intervals, and a default duration of one hour. Subsequently, the following additional information is asked depending on the activity category and age group:

- Intensity (for playing: active/calm/I don’t know; for screen use: watching/playing calmly/playing actively/I don’t know);
- Whether the child was restrained (yes/no/I don’t know);
- Posture (lying on tummy/lying on back/lying on side/sitting with support/sitting without support/standing with support/standing without support/being carried/changing posture/ I don’t know);
- Location (at home indoor/at home outdoor/childcare indoor/childcare outdoor/neighborhood indoor/neighborhood outdoor/other);
- Who were present (one or more other children/one or more other adults/my child was alone/other/I don’t know);
- Type of device screen use (television/tablet/smartphone/game console or computer/other/I don’t know).

**Fig. 3:**
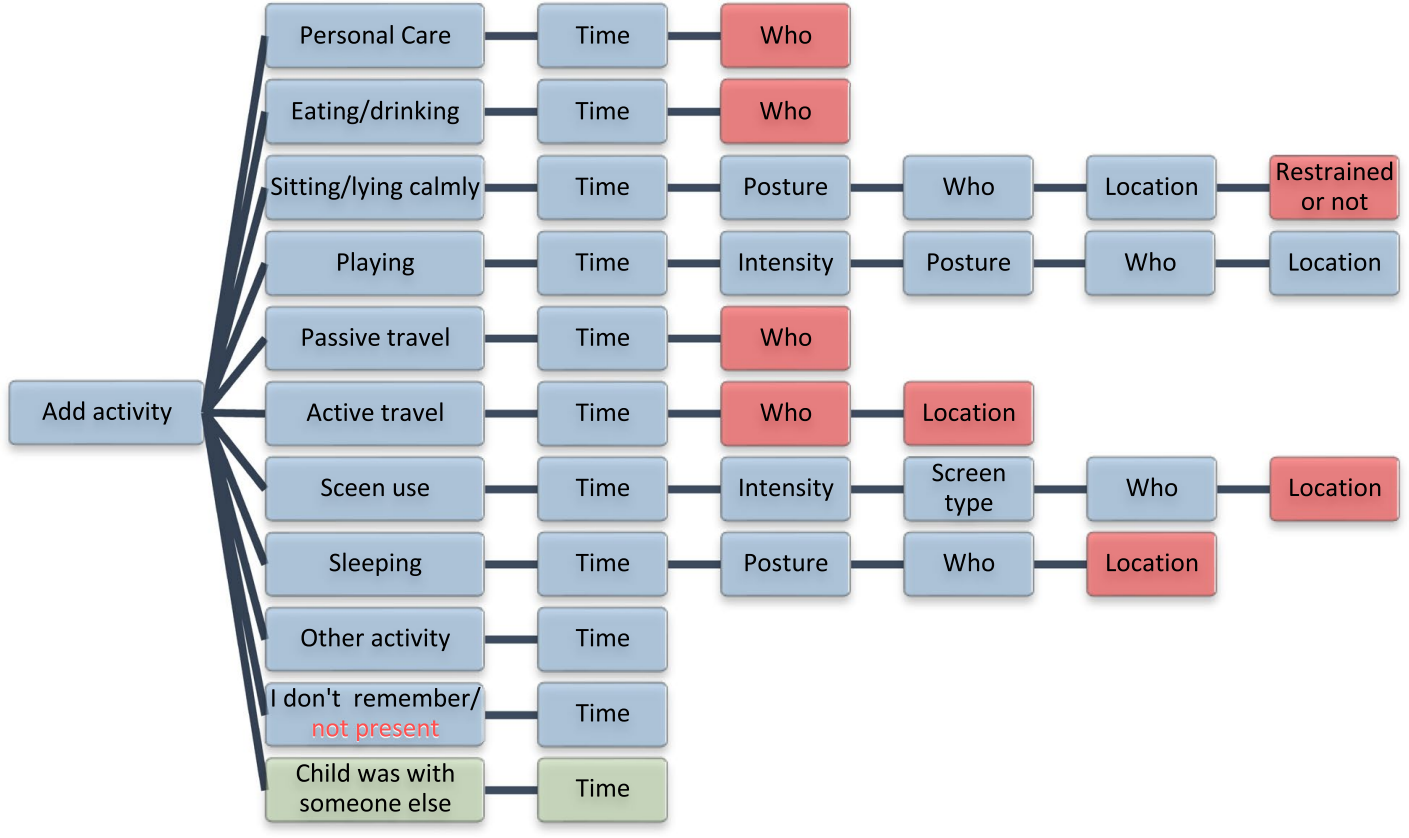
Sequence of follow-up questions asked per activity category in the My Little Moves app. Red: removed after the first content validity study (step 3); Green: added after the first content validity study (step 3)

Figure 3 shows an overview of the activity categories and the follow-up questions providing the additional information for each activity category.

### Step 3: Content validity of first version of My Little Moves

#### Participants

Seventeen parents and 7 researchers signed informed consent, of which one parent did not meet the eligibility criteria (i.e., age child older than 4 years), and two parents and one researcher were not able to attend the planned (focus group) interview. Therefore, 14 parents and 6 researchers participated in the first content validity study. Table 4 shows the socio-demographic characteristics of the participants. The majority of parents were highly educated, with 11 parents having a master’s degree. Two online focus groups were held with parents (*n* = 2–3 per group), and two focus groups with researchers (*n* = 2–3 per group). Eleven individual interviews were held with parents, and one with a researcher.

**Table 4:** Socio-demographic characteristics of participants in the content validity study of the first- and adapted version of the My Little Moves app

Version My Little Moves app	First version		Adapted version
**Group**	**Parents**	**Researchers**	**Parents**
**Total number**	14	6	5
**Age group children**			
0–6 months	0	NA	0
6–12 months	2		1
12–24 months	10		0
24–36 months	2		2
36–48 months	1		2
**Mean age ± SD (years)**	32.4 ± 1.8	NA	31.0 ± 5.4
**Gender**			
Female / Male	11 / 3	6 / 0	5 / 0
**Highest educational level**			
SED or VOC ED	1	NA	5
Bachelor’s degree	2		0
Master’s degree	11		0
**Country of birth**			
Netherlands	14	6	5
Other	0	0	0
**Expertise**^**a**^			
Developing measurements instrument	NA	4	NA
Evaluating measurements instruments		5	
Physical activity in young children		3	
Sedentary behavior in young children		1	
Sleep in young children		1	
**Mean experience current occupation ± SD (years)**	NA	6.4 ± 4.2	NA

*Abbreviations: SD* standard deviation, *SED S*econdary education, *NA* not applicable, *VOC ED* Vocational education

^a^Some researchers had expertise on multiple of these subjects

#### Feasibility and user-friendliness

Although most parents did not test the app for seven days, 11 of the 14 parents (78%) considered filling in the app for seven consecutive days feasible. However, some parents also indicated that reporting young children’s activities was challenging: *‘Moments are often short, so you have to fill in a lot’ (parent 9).* Parents also indicated that if they had to fill in the app for a week, data would be incomplete because the child was at the daycare center one or more days a week. Filling in the app took parents between 10 and 30 min per day. Some parents and researchers indicated that it might be difficult to remember at what age a specific motor milestone was achieved.

When using the app for the first time, the parents and researchers understood how to create an account and how to add activities to the timeline: ‘*I love how you add the activities’ (parent 1), ‘I found it easy. I didn’t have to think much or look for what to do’ (parent 3)*. Most participants liked the design and layout of the app. However, there were some points for improvement regarding the user-friendliness. One of the main inconveniences was that the password could not be reset. Parents also disliked having to log in again after automatic logout. In addition, multiple parents and researchers mentioned that the location of the logout button at the contact page was illogical. However, this was not considered a major problem as most people would rarely use this. Another frequently mentioned inconvenience was that entered activities could not be modified, e.g. if the activity started at 15.00 instead of 15.30. In such cases, the activity would have to be deleted and added again. Moreover, several parents indicated that the default duration of an activity should be reduced to less than one hour, to limit unnecessary scrolling, as most of young children’s activities take less time. Also, when selecting the time, certain smartphones vibrated, which was considered annoying: ‘*As a parent, this vibration would personally drive me crazy if I had to fill in activities for the whole day.’ (researcher 3).* Lastly, parents were sometimes unsure whether they pressed the button they meant to press because the screen immediately changed to the next follow-up question without a confirmation.

#### Comprehensiveness

Most participants indicated that the app included all relevant activity categories. A few parents suggested splitting up the category ‘I don’t remember/not present’, as they felt they do not reflect the same thing. In addition, one parent indicated that ‘not present’ sounds a bit negative, and therefore could be reworded, for example to ‘child was with someone else’. Some parents indicated that they missed an activity such as helping with household activities or doing chores*: ‘A small child often imitates things, for example cleaning, and I couldn’t enter this. You may have to enter this as playing, but that is not listed in the examples.’ (parent 5*). After choosing the category ‘other activity’, some parents would like to have the possibility to specify which activity their child did. In addition, for multiple categories other activity examples were suggested for specific age groups, for example for children aged 1–2 years, adding ‘breastfeeding’ to the category ‘eating and drinking’ and ‘sitting in a bicycle seat’ to the category ‘passive transport’*.* Moreover, additional answering options for follow-up questions were suggested, for example adding the option ‘both other children and adults’ to indicate who were present at the activity.

#### Relevance

The activity categories were generally considered relevant. A few parents mentioned that they rarely used the category ‘sitting/lying calmly’: *‘This only really happens at 0–6 months. Any older child that can sit still for 10 min is either sick or asleep.’ (parent 1).* However, other parents indicated the relevance of this category. One of the researchers indicated that the category ‘other activity’ could be removed, as this would only result in missing data*.* However, other researchers and parents argued that they would keep this category. Multiple parents and researchers suggested removing some of the activity examples as these did not add to the clarity (e.g., for playing). In addition, the question ‘Who were present at the activity?’ was considered irrelevant by multiple parents, as very young children rarely do something without the presence of an adult. Also, they questioned the relevance of this information. With regard to the location of the activity, parents and researchers questioned the necessity of other answering options than ‘outdoor’ and ‘indoor’, e.g. ‘at home’ or ‘in the neighborhood’.

#### Comprehensibility

In general, the participants understood how the app worked and how to add activities. Although difficult to report, the questions on motor milestones were generally considered comprehensible. However, there were some issues regarding the comprehensibility. For example, some parents and researchers had difficulties understanding the difference between (active) playing and active transport, e.g., it was not clear which category to choose for activities such as crawling or running while playing. In addition, parents sometimes considered it difficult to report whether playing was active or passive: *‘A child of 1.5 years, who runs from one side of the room to the other, then sits down to read a book, and then runs up and down the room again. Is that passive or active?’ (parent 1)*. Moreover, parents indicated that children often do multiple activities at the same time, such as watching television while eating, or sleeping while sitting in the car. This complicates reporting activities as it is then unclear which activity category to choose. Lastly, both parents and researchers indicated that some texts in the app could be simplified, and gave multiple suggestions to increase comprehensibility e.g. ‘daily overview’ instead of ‘timeline’ to reflect the meaning of that specific page.

### Step 4: Adaptation of My Little Moves

Multiple limitations and suggestions for improvement were adopted in the second version of the My Little Moves app. Figure 3 shows the follow-up questions that were considered irrelevant (in red) and the questions that were added/rephrased, e.g. the category ‘I don’t remember/not present’ was split up, and rephrased as ‘I don’t remember’ and ‘child was with someone else’. We decided not to add an open input field to the category ‘other activity’ to specify the activity, for two reasons: first, because this would add an additional interpretation step in the analysis, and second, for data privacy reasons to prevent that parents would enter personal identifiable information. To increase comprehensibility, we made multiple textual changes throughout the app, e.g. for the follow-up question on the intensity of playing (i.e., ‘Did your child play *mostly* actively or calmly?’). To further improve the comprehensibility of activity categories (e.g., for active transport or playing) other examples were included. Moreover, we added explanations under the ‘frequently asked questions’ in the app, e.g. how to deal with situations in which the child is doing two activities at the same time: choose the activity that in your opinion best suits with what the child itself is doing (e.g., child sleeps in the stroller, the activity is then sleeping). In addition, answering options identified as less relevant were removed (e.g., answering options for location of activity were reduced to ‘indoors’ or ‘outdoors’) and missing options were added (e.g., ‘both other children and adults’ being present at the activity). Moreover, the default time for the activity duration was changed to half an hour, and the period after which app-users were automatically logged out was extended to two weeks. Also, the adapted app more clearly showed which button was pressed by highlighting it.

Due to a limited budget, we unfortunately could not resolve all indicated limitations of the app. For instance: resetting or retrieving the password of participants, and modifying reported activities could not be resolved. However, we made textual changes at the login page to emphasize the importance of remembering or storing the password. Additional file 2 presents screenshots of the adapted version of the app (version 1.1.0).

### Step 5: Content validity of adapted version of My Little Moves

Five parents signed informed consent to test the adapted version of the My Little Moves app, and participated in individual interviews. Table 4 shows the socio-demographic characteristics of the participants. In general, parents were positive about the user-friendliness, relevance, comprehensiveness and comprehensibility of the app. The most frequently mentioned limitations were similar to those in the first content validity study, but could not be resolved in step 4 (e.g. due to a limited budget). For example, the impossibility of the app to retrieve the password of participants, not being able to specify the activity in the ‘other activity’ category, and the illogical location of the logout button. Two parents additionally mentioned that the large number of follow-up questions limits the feasibility of the app. The most important newly mentioned limitation was that some parents did not fully understand the difference between active and passive transport. In addition, as the daily time-use overview only shows the entered activity categories, two parents would have liked to see the additional information (from follow questions) of the entered activities in this overview, e.g. the intensity or location.

## Discussion

The aim of this study was to design a mobile app to assess 24-h movement behaviors in 0–4-year-old children while involving end-users and other relevant stakeholders in the development and content validity evaluation. The My Little Moves app consists of a time-use diary in which parents can proxy-report the activities of their child for seven consecutive days. In addition to the duration and timing of activities, the app also collects information on the type of activity, its intensity, posture, individual(s) present, and location. The concept mapping and content validity studies resulted in a tailored tool to assess 24-h movement behaviors in young children.

The My Little Moves app offers several novel features when compared to other tools for proxy-reporting young children’s movement behaviors, such as the recently developed Movement Measurement in the Early Years (MoveMEY) tool [33] or Movement Behaviour Questionnaire (MBQ) [34]. First, it is a mobile app instead of a paper-based tool, with minimal software requirements for Android or iOS. Second, it includes a time-use activity diary for the assessment of all 24-h movement behaviors (physical activity, sedentary behavior and sleep). Third, it covers the full age range of 0–4-year-old children, while the app adapts both to age and motor developmental stage of the child, thereby providing tailored questions, examples of activities, and answering options.

Compared to similar online tools for older children to report their 24-h movement behaviors such as MyDailyMoves [30] or My E-Diary for Activities and Lifestyle (MEDAL) [35, 36], the My Little Moves app has some notable differences. First, it is a mobile app, instead of a web-based app. This makes the My Little Moves app convenient to use on a mobile phone or tablet and therefore more feasible to use throughout the day. Second, while MyDailyMoves asks children to recall the activities of the previous day, in MEDAL and the My Little Moves app parents are able to enter the activities in real-time, thereby reducing recall bias as much as possible. Last, as MyDailyMoves and MEDAL are tools for older children, the output includes an intensity rating for each activity based on the rating of the perceived exertion and the Metabolic Equivalent (MET) values from the Compendium of Energy Expenditure for Youth [37]. Determining the intensity of physical activities is complicated in the current age group as corresponding MET intensity levels are missing [37, 38]. Therefore, in the My Little Moves app we collect information on the intensity of activities by asking whether the activity was active or calm (e.g., for playing) and/or in what posture the activity was performed (e.g., while standing, sitting, or lying down).

It is challenging to proxy-report young children’s 24-h movement behaviors. First, young children’s activities are sporadic and intermittent, and are rarely done for a continuous period of time [15]. Therefore, it can be argued that it is unrealistic to expect that proxy-report tools can be used to accurately assess 24-h movement behaviors. To improve accuracy, we opted for a time-use diary format, whereas most previous tools rely on recalling the duration and/or frequency of engaging in different activities, such as in the past week or a typical week [19]. This choice was based on previous findings indicating that a time-use diary format contributes to the accuracy of reported activities [19, 31]. For example, a concurrent validity study of Zhang et al. (2022) showed that a paper-diary was more accurate in reporting tummy time in infants than a questionnaire recalling a typical day [31]. A diary format reduces the chance of over- and/or underestimation of time spent in different activities. A second challenge when proxy-reporting young children’s 24-h movement behaviors is that children are not always within the sight of their parents, for example when they are at a childcare center. This could lead to incomplete data. Although the app could potentially be used by multiple caregivers per child and on multiple devices, this is not possible within the current version of the app. Last, while the majority of parents expressed confidence in their ability to complete the app for seven consecutive days, the time commitment for completing the app (i.e., 10–30 min per day) may prove burdensome for some parents. Since, in the present study, parents were only requested to complete the app for a minimum of one day, we are unable to confirm the feasibility of completing the app for multiple days. It is difficult to compare the feasibility of the My Little Moves app with other proxy-report tools for this age group, as the feasibility of these tools has rarely been investigated [19, 20]. A recent study using a parent-report 3-day time-use diary at three time points to examine movement behaviors in infants, showed an average completion rate of > 95%, and indicated that the tool was feasible [39, 40]. This is promising for the use of tools with a time-use format in early childhood, including the My Little Moves app.

### Strengths & limitations

A major strength of this study is the involvement of parents and professionals working with young children in the concept mapping study used for the development of the app, as they provide the lived experience regarding activities of young children. Another strength is the involvement of both parents and researchers in evaluating the user-friendliness, comprehensiveness, relevance and comprehensibility of the app. In addition, we conducted two content validity studies following the COSMIN methodology which further strengthens our study [22].

A limitation of our study was that most parents who participated in step 1 (i.e., concept mapping) and step 3 (i.e., content validity of first app version) were highly educated. Therefore, in step 5 (i.e., content validity of adapted app version), we included only parents with a maximum educational level of secondary education or vocational education, but this sample was small. Another limitation is that the My Little Moves app is a Dutch app, so only Dutch-reading participants were included. In addition, in steps 3 and 5, parents were asked to complete the app for at least one day, which limits our ability to confirm the feasibility of completing the app for multiple days. Last, due to limitations in available time and budget we were not able resolve all mentioned limitations of the app.

### Future studies and recommendations

The next step for future studies is to examine the reliability and construct validity of the My Little Moves app to further evaluate the quality of the tool. However, evaluating the construct validity is challenging as a gold standard for assessing 24-h movement behaviors in this age group is lacking [13]. To evaluate the extent to which data obtained with the My Little Moves app reflects all 24-h movement behaviors, as a next step, we will investigate comparability of the activities assessed with the app and the corresponding accelerometer output in children aged 0 to 4 years old. In addition, we will examine the requirements for obtaining reliable data, e.g. the minimal number of hours and days that parents have to complete the My Little Moves app to obtain representative data on their children’s 24-h movement behaviors. Subsequently, we recommend future studies to explore parents’ willingness and needs in terms of compensation to complete the app for this required period.

In addition, future studies that aim to design an app, are recommended to reserve sufficient budget for multiple adaptation rounds. For potential further adaptation of the My Little Moves app, we recommend enabling the following: 1) reset or retrieve the password of app-users, 2) allow multiple caregivers to use the app (each from their own device) to get a more complete picture, and 3) enter multiple simultaneous activities (e.g., screen use while playing). Furthermore, we recommend to translate the app in multiple languages, and make it accessible for other research projects. Before using the (translated) app in other countries, we recommend repeating the content validity study in that particular country. We recommend future content validity studies to examine the feasibility, user-friendliness, comprehensiveness, relevance and comprehensibility in a larger and more diverse sample, who use the app for at least seven days.

Last, as it is difficult to accurately proxy-report all children’s activities, we recommend future studies, if feasible, to use accelerometers alongside proxy-report tools such as the My Little Moves app, as both instruments could provide complementary data [20]. In future studies, we will further examine how data obtained from the My Little Moves app and accelerometers can complement each other.

## Conclusions

Involving end-users and other relevant stakeholders in the development and content validity studies of the My Little Moves app resulted in a tailored tool to assess 24-h movement behaviors in children aged 0–4 years. This app is promising for monitoring 24-h movement behaviors in large samples of young children. In future studies, we will further evaluate the measurement properties of the app.

### Supplementary Information

**Additional file 1. **Concept mapping clusters.

**Additional file 2. **Screenshots My Little Moves app.

## Data Availability

The data that support the findings of this publication are available from the corresponding author upon reasonable request.

## Abbreviations

App: Application
COSMIN: COnsensus-based Standards for the selection of health Measurement INstruments
ECEC: Early childhood education and care
MBQ: Movement Behaviour Questionnaire
MEDAL: My E-Diary for Activities and Lifestyle
MET: Metabolic Equivalent
MoveMEY: Movement Measurement in the Early Years
