# Supplementary material for: Development and content validity of an application to assess 24-hour movement behaviors in 0–4-year-old children involving end-users and key stakeholders: the My Little Moves app
Source: Int J Behav Nutr Phys Act. 2024 Jan 2;21:2. doi: 10.1186/s12966-023-01552-9 (PMC10763169; doi:10.1186/s12966-023-01552-9)

My Little Moves app version 1.1.0

Login screens.
From left to right: screen to enter research code; screen to create a password; screen to login with created password.

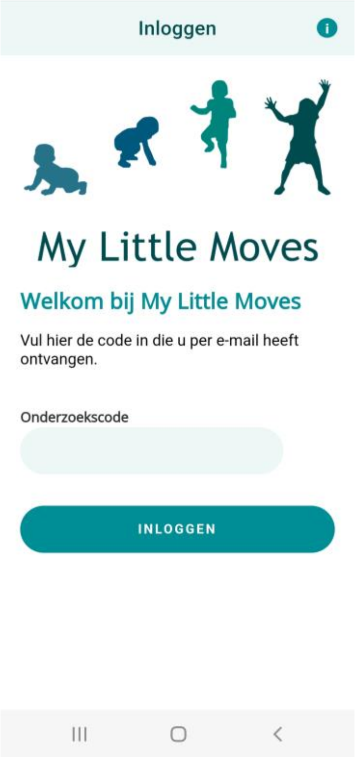

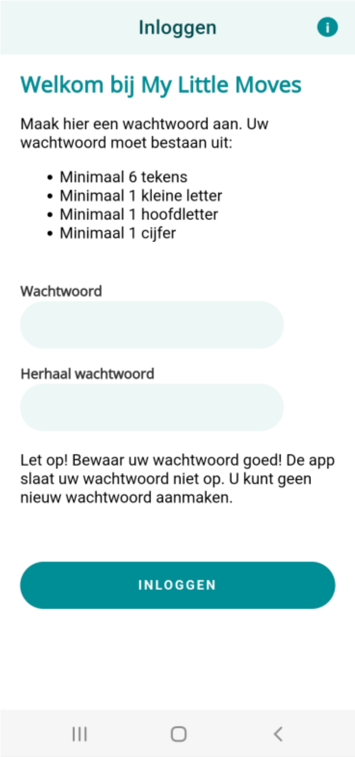

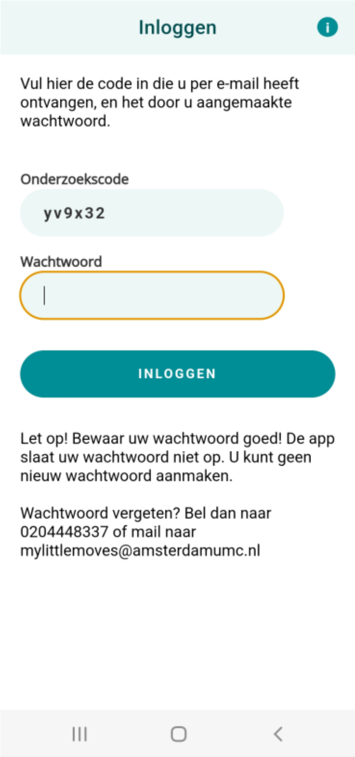

Screen start measurement period.
Information about the My Little Moves project and app. With pressing the green button, the 7 day-measurement period will be started.

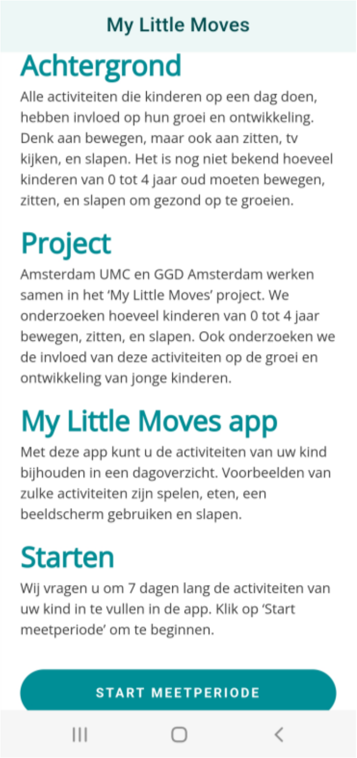

Screens intake questionnaire.

From left to right: intake question to assess the child's age group (i.e., 0-6 months, 6-12 months; 1-2 years, 2-3 years or 3-4 years); intake question to assess the child's motor development (i.e., whether child is able to crawl at least 1.5 meter).

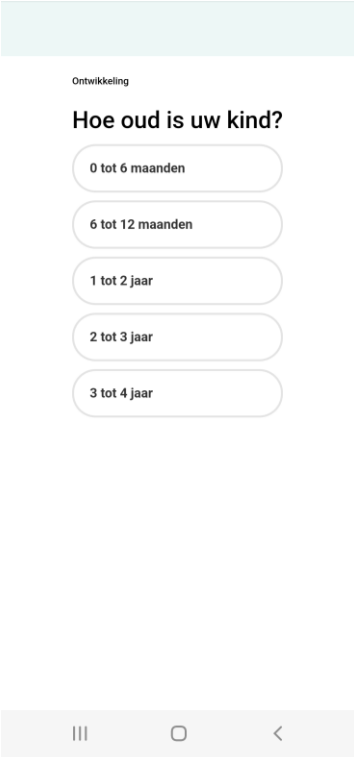

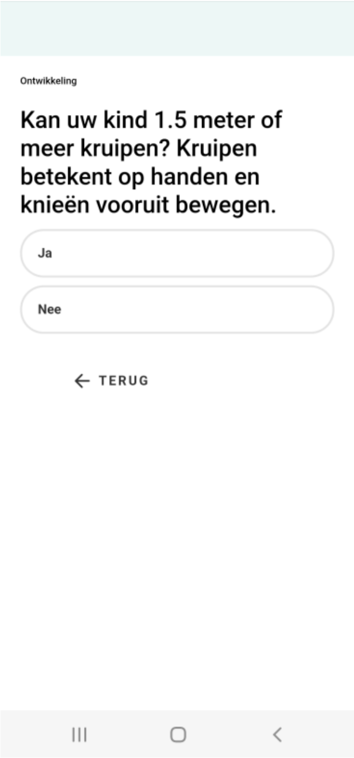

Screens main tabs.

From left to right: daily time-use overview; information about the research project; contact information and frequently asked questions.

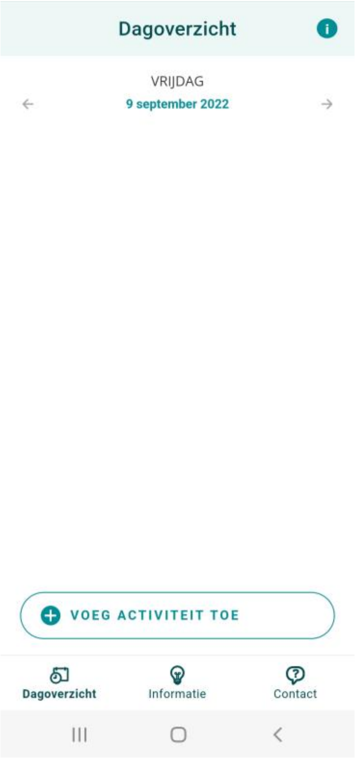

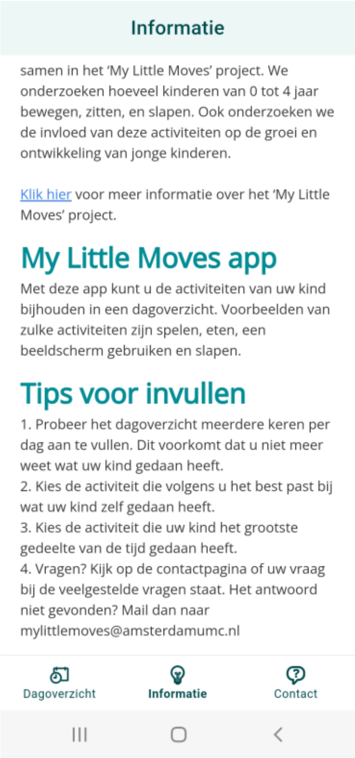

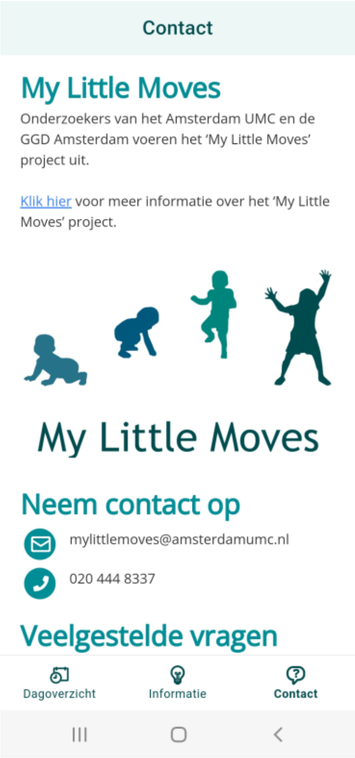

Screens of adding an activity to the time-use overview.
From left to right and top to bottum: choosing an activity category, with age-appropriate activity examples for clarification of each category; selecting an activity category; selecting the time of the activity; follow-up question for the activity category ‘playing’: ‘*Did your child play mostly actively or calmly?’;* follow-up question for the activity category ‘playing’: ‘*Where was this activity*?’; entered activities in the daily time-use overview

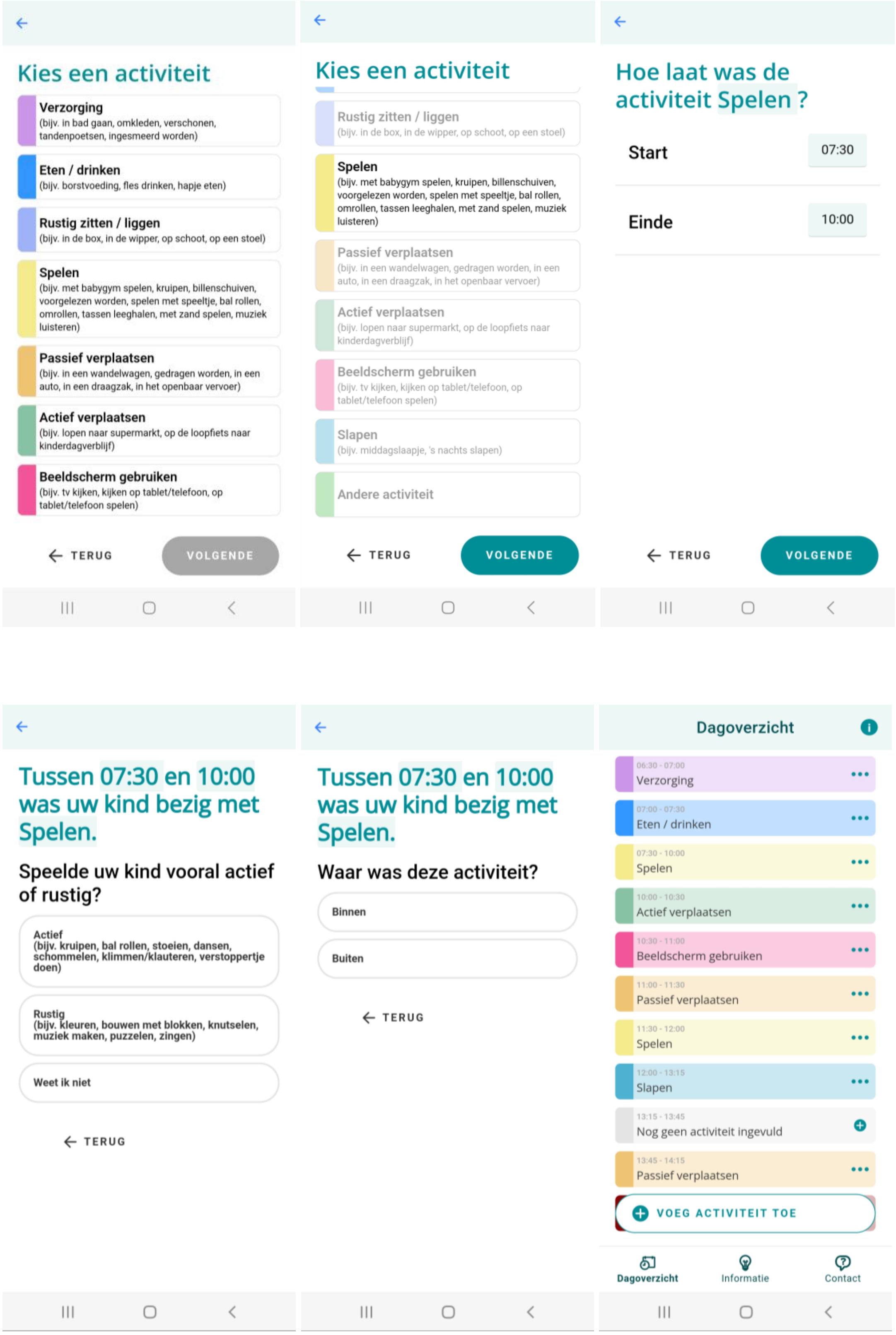

Supplement: Supplementary file 2 — Additional file 2. Screenshots My Little Moves app. [file 12966_2023_1552_MOESM2_ESM.pdf]
